# Supplements, homepage, newsletter and mission of the journal

**DOI:** 10.1590/S1980-57642009DN20400001

**Published:** 2008

**Authors:** Ricardo Nitrini

**Affiliations:** Editor-in-Chief

**Dementia & Neuropsychologia** reaches the end of its second year of publication, jostling to find its place among the high quality journals of its kind, while abiding by the stringent rules of the indexation systems. This issue includes three papers from abroad, testimony to the faith which members of the international scientific community place in this journal’s future.

Besides its four regular issues for the year, one supplement was also published in 2008, containing the abstracts of the Sixth Congress of Rehabilitation promoted by the Brazilian Society of Neuropsychology. The opening of the journal to carry abstracts of meetings in its areas of interest has been a important function of the journal from its outset. Abstracts of the Brazilian Congress on Brain, Behavior and Emotions were also published both in 2007 and 2008, while abstracts of the Brazilian Meeting on Alzheimer’s Disease and Related Disorders as well as the Second International and Ninth Brazilian Congress from the Brazilian Society of Neuropsychology were published in 2007. It is the intention of the editorial board to continue in this vein, and to carry supplements or abstracts of international meetings.

**Dementia & Neuropsychologia** is nearing implementation of its home page, a significant achievement for the journal. It is available at: demneuropsy.com.br. The home page will facilitate the search for papers using key words, furnish information on number of visitors to each article, and follow the interest of the scientific community in **Dementia & Neuropsychologia**. A newsletter system, a key strategy to promote and disseminate recently published papers, is now being prepared. The goal is for this newsletter to reach a vast array of neurologists, psychologists, psychiatrists, speech therapists, geriatricians and basic scientists.

The **Instructions to authors** have been changed to include the mission of the journal as follows: **Dementia & Neuropsychologia** is a quarterly journal dedicated to publishing research in cognitive and behavioral sciences, focusing on clinical epidemiology, basic and applied neurosciences, and cognitive tests devised or adapted for populations with heterogeneous educational and socioeconomic backgrounds. **Dementia & Neuropsychologia** is particularly involved in publishing research relevant to developing countries, and also seeks to disseminate reviews and case reports that are important contributions to neurological, psychiatric, geriatric, neuropsychological, speech therapy, and related fields. The editorial board considers that with this mission **Dementia & Neuropsychologia** clearly states that it is not simply another journal, but that it has a specific role and niche among the journals in the area. The editorial board is also confident that these new achievements will pave the way for indexing of the journal in the forthcoming months.

To tie up the edition of this issue and on behalf of the editorial board, I wish to express our gratitude to the scientific community, not only within Brazil, but also across all Latin American countries and beyond, who have generously submitted papers and invested time in the peer-reviewing process. I also thank all our colleagues from back office, the copydesk, printers and English language consultation and extend to them, our readers and sponsors, our wishes for a Prosperous and Happy 2009.

**Ricardo Nitrini** Editor-in-Chief

